# Genetic characteristic of class 1 integrons in proteus mirabilis isolates from urine samples

**DOI:** 10.1051/bmdcn/2017070202

**Published:** 2017-06-14

**Authors:** Chih-Ming Chen, Chih-Ho Lai, Hwa-Jene Wu, Lii-Tzu Wu

**Affiliations:** 1 Division of Infectious Disease, Department of Internal Medicine, Tungs’ Taichung MetroHarbor Hospital Taichung 433 Taiwan; 2 Department of Health Food, Chung Chou University of Science and Technology Changhua 510 Taiwan; 3 Graduate Institute of Biomedical Sciences, Department of Microbiology and Immunology, College of Medicine, Chang Gung University Taoyuan 333 Taiwan; 4 Molecular Infectious Disease Research Center, Department of Pediatrics, Chang Gung Children’s Hospital and Chang Gung Memorial Hospital Taoyuan 333 Taiwan; 5 Graduate Institute of Basic Medical Science, School of Medicine, China Medical University Taichung 404 Taiwan; 6 Department of Medical Research and Department of Laboratory Medicine, China Medical University Hospital Taichung 404 Taiwan; 7 Department of Nursing, Asia University Taichung 413 Taiwan; 8 Department of Clinical Laboratory, Jen-Ai Hospital Taichung 412 Taiwan

**Keywords:** ERIC, ESBL, Integron, *Proteus mirabilis*

## Abstract

Background: *Proteus mirabilis* is an opportunistic pathogen, commonly associated with complicated urinary tract infections (UTIs). UTIs caused by multidrug-resistant *Proteus mirabilis* have increased worldwide. Multidrug-resistance of Gram-negative enteric bacteria is usually associated with class 1 integrons.

Purposes: To investigate the prevalence and characterize gene cassettes of class 1 integrons in multidrug-resistant *P. mirabilis*

Methods: From 2006 to 2008, 314 *P. mirabilis* isolates from urine were collected from a regional teaching hospital. Antimicrobial resistance of the isolates was determined by disk diffusion methods. The phenotypic confirmatory test of extended-spectrum β-lactamase (ESBL) production was performed as described in the Clinical and Laboratory Standards Institute (CLSI) guideline. The genetic organization of the class 1 integron cassettes was investigated by PCR, cloning, and sequencing of the regions surrounding these genes.

Results: Seventy-nine (25%, 79/314) *P. mirabilis* isolates were ESBL-producing and most ESBL-producing *P. mirabilis* were positive for *bla*_CTX-M_. Class 1 integrons were presented in 76 isolates (24.2%, 76/314), and were more frequently found in ESBL-positive (55/79, 70%) than ESBL-negative (21/235, 8.9%) *P. mirabilis* isolates. The most prevalence of the cassettes encoded resistance genes were aminoglycoside *(aac(6’)-Ib, aacA7, aadAl, aadA2,* and *aadAla),* trimethoprim *(dfrAl* and *dfrA12)* and chloramphenicol *(catB3* and *cmlA6).* The most prevalent cassette of *dfr12-orfF-aadA2* was found in 49 isolates. The cassette array *aadB-catB3-oxa10-aadA1* was first found in *P. mirabilis.* The enterobacterial repetitive intergenic consensus (ERIC)-PCR fingerprinting patterns were detected in these 76 integron positive *P. mirabilis* isolates and belonged to 8 profiles.

Conclusion: This study investigated the prevalence and characterized gene cassettes of class 1 integrons in MDR *P. mirabilis* isolates from urine samples. The frequency of gene cassettes in *P. mirabilis* were partially by clonal spread of the carriers and the results could provide information for effective antimicrobial therapy and infection control.

## 1. Introduction

The antimicrobial genes frequently located on plasmids, transposons and integrons, lead to the rapid dissemination and treatment problem. Class 1 integrons has been proven the most common integron type present in clinical isolates of Gram-negative enteric bacteria mostly in *Enterobacteriaceae, Pseudomonas aeruginosa* and *Acinetobacter baumannii* [1]. Class 1 integrons usually associated with multidrug-resistance due to their ability to incorporate or excise one or more antimicrobial resistance gene cassettes.These genes can be integrated in the form of cassettes by three key components: the *inti* gene for recombination, the *attl* site for primary recombination known as 59 base elements, and a promoter Pc that directs expression of the cassette-encoded genes [2]. As the consequence of different insert genes, the variety of linked backbone structures of both cassettes and integrons also suggests an important role of these elements in adaptive evolution [3].

*Proteus mirabilis* is an opportunistic pathogen, commonly associated with complicated urinary tract infections (UTI) among patients with urolithiasis and long-term urinary catheterization in both community and healthcare settings. Infection by multidrug- resistant (MDR) *P. mirabilis* infections has increased worldwide in the past few years [4–7], due to its rapid acquisition and dissemination of a wide variety of antibiotic resistance genes, as well as other members of the *Enterobacteriaceae* family express β-lactamase. The β-lactam resistance patterns of the *P. mirabilis* isolates have reported production of various class extended-spectrum β-lactamases (ESBLs) and AmpC-type cephalosporinases [6, 8–10]. Previously, we reported that *P. mirabilis* isolates from Taiwan were susceptible to imipenem, ceftazidime, cefepime (MICs ≤ 0.5 μg/ml) but exhibited high level resistance to cefotaxime (MIC > 256 μg/ml) [11]. A high level resistance of cefotaxime is mainly mediated by co-existence of AmpC enzymes with CTX-M-type β-lactamases among *P. mirabilis* in Taiwan [6, 11].

The spread of ESBLs represents a serious threat to the management of infectious diseases that restricts therapeutic options of antimicrobial uses. The prevalence of integrons and characterized gene cassettes in Gram-negative bacteria integron-associated multidrug resistance have been investigated [12–15]; however, it is seldom addressed in *P. mirabilis*. In this study, a comparison of integron-carrying and non-integron-carrying MDR *P. mirabilis* isolated from urine was made to assess the differences in their antimicrobial susceptibility and clonal dissemination.

## 2. Materials and methods

### 2.1. Organisms

From 2006 to 2008, non-duplicate *P. mirabilis* isolates with ampicillin resistance (n = 314) of patients with urinary tract infection were collected from a 746-bed, tertiary care regional teaching hospital in middle Taiwan (Jen-Ai hospital, Taichung). These isolates were identified on the basis of routine microbiologic methods and confirmed using the VITEK system (BioMerieux Vitek Inc, Hazelwood, MO, USA). *Escherichia coli* DH5a was used as the host for transformation experiments.

### 2.2. Antibiotic resistance

Identification the resistance phenotype of the gene cassettes within the integrons was undertaken by disc diffusion test according to the recommendations of the Clinical and Laboratory Standards Institute (CLSI). The combination-disk (Becton Dickinson Microbiology Systems, Cockeysville, MD, USA) synergy tests were performed to detect ESBL and additional AmpC β-lactamases phenotype for all the collected isolates [16, 17]. All disks were purchased from Becton Dickinson Microbiology System. The MICs of antimicrobial agents were determined by the agar gradient diffusion technique (E-test and ESBL Screen, AB BIODISK, Solna, Sweden) according to the manufacturer’s instructions. The ESBL phenotype was confirmed by a reduction of ≥ 3 doubling dilutions for MICs of either cefotaxime or ceftazidime in the presence of clavulanic acid [17]. Control strains included *E. coli* ATCC 25922 and *Klebsiella pneumoniae* 700603.

### 2.3. Integron detection and typing, and PCR amplification of β-lactamase-encoding genes (*bla*)

The genetic organization of the class 1 integron cassettes was investigated by PCR, cloning, and sequencing of the regions surrounding these genes. Primers p-1 (5′-CGGATGAAGGCACGAAC-3′) and p-2 (5′-AAGCAGACTTGACCTGA-3′) were used for class 1 integron detection [18]. Polymerase chain reaction (PCR) detection and sequencing of *bla* genes coding for the TEM, SHV, CTX-M and CMY enzymes were performed as described previously [11]. PCR conditions for all these genes were 3 min at 94 °C; 30 cycles of 1 min at 94 °C, 1 min at 55 °C, and 2 min at 72 °C; and finally, 7 min at 72 °C. The amplicons were revealed by electrophoresis on a 1.0% agarose gel with 0.5 × TBE (Tris-borate-EDTA) running buffer and a subsequent exposure to UV light in the presence of ethidium bromide. The amplicons were purified with PCR clean up kits (Genemark, Taichung, Taiwan) and were sequenced by an ABI PRISM 377 sequencer analyzer (Applied Biosystems, Foster City, CA, USA). Sequence analyses were performed online at the National Center for Biotechnology Information website (http:// www.ncbi.nlm.nih.gov). The phylogenetic relations of int1-positive *P. mirabilis* were analyzed using enterobacterial repetitive intergenic consensus (ERIC)-PCR with primer ERIC2 (5′-AAG-TAAGTGACTGGGGTGAGCG-3′) [19]. The following conditions of amplification were used: denaturation for 5 min at 94 °C; 40 cycles of 45 s at 94 °C, 1 min at 52 °C and 5 min at 72 °C; and a final extension step of 10 min at 72 °C. All PCR products were separated by electrophoresis in 1% agarose in 0.5 × Tris/acetate/ EDTA buffer for 1 h at 100 V. The generated fingerprints were compared visually.

### 2.4. Amplicon cloning and sequence

Isolates yielding two and three amplicons of difference sizes were inserted into a cloning vector (pGEM-T easy) according to the instructions for a pGEM-T easy cloning kit (Promega, cooperation USA), and *E. coli* DH5a was transformed with the recombinant plasmid. The sequence of the insert was verified by nucleotide sequencing.

## 3. Results and discussion

### 3.1. Antimicrobial susceptibility and ESBL survey

MIC distributions and resistance rates of tested antimicrobial agents are shown in Table 1. Except ceftazidime and meropenem, integron-positive isolates have significantly higher resistance rate (*p* < 0.05) than integron-negative isolates. Eighty-seven isolates (27.7%) presented a multi-resistant phenotype (resistance to three or more antimicrobial families) and 25 isolates (8.0%) exhibited resistance to at least five different families of antimicrobial agents (data not shown). Moreover, 79 (25.1%) of the 314 isolates exhibited a positive ESBL test. Among 79 ESBL-producing *P. mirabilis* isolates, 55 (69.6%) were class 1 integron positive (Table 2). By PCR and nucleotide sequencing, we detected the presence of only CTX-M-14 in 70 isolates, only CTX-M-3 in 2 isolates, and both CTX-M-14 and CTX-M-3 in 7 isolates. Furthermore, all of the 79 isolates confirmed 100% identity with bla_TEM-1_ and no amplicons were observed for bla_SHV_ and plasmid-mediated AmpC β-lactamases bla_CMY_ genes. CTX-M-3 and CTX-M-14 were the most common CTX-M variants in this study as were reported previously in a survey in 2009 in middle Taiwan [20], but different as Enterobacteriaceae coding CTX-M-15 in Asia-Pacific region [21]. Although ESBL-producing *P. mirabilis* isolates coproducing CTX-M (27.7% in this study) have been shown to be major pathogens for urine infection, the trends of CTX-M variants or other ESBLs resistance should be carefully monitored.

**Table 1:** Susceptibility testing results of 76 *intll-positive* and 238 *intll-negative Proteus mirabilis* isolates.

	intl1-positive isolates (*n* = 76) ESBL positive n = 55		intl1-negative isolates (n = 238) ESBL positive n = 24	
Antibiotic	MIC (μg/*ml*) (no. of isolates)	Resistance	MIC (μg/*ml*) (no. of isolates)	Resistance
Amoxicillin-clavulanic acida	16 (4), ≤ 8 (72)	5.2%	16 (2), ≤ 8 (236)	0.1%
Piperacillina	≥ 256 (75), 64 (1)	98.7%	≥ 256 (35), 32 (203)	14.7%
Cefotaximea	≥ 256 (29), 64 (13), 16–32 (12), ≤ 2 (22)	71.1%	≥ 256 (10), 64 (7), 16-32 (7), ≤ 2 (214)	10.1%
Ceftazidime	2(1), ≤ 0.5 (75)	1.3%	≤ 0.5 (238)	0%
Cefepimea	32 (1), 4 (3), ≤ 2 (72)	5.3%	4 (1), ≤ 2 (237)	~0%
Cefoxitina	16–32 (3), ≤ 8 (73)	3.9%	16 (1), ≤ 8 (237)	~0%
Meropenem	≤ 0.5 (76)	0%	≤ 0.5 (238)	0%
Gentamicina	≥ 16 (66), ≤ 2 (10)	86.8%	≥ 16 (21), ≤ 2 (217)	8.8%
Amikacina	≥ 16 (65), ≤ 2 (11)	85.5%	≥ 16 (17), ≤ 2 (221)	7.1%
Ciprofloxacina	≥ 4 (28), ≤ 1 (48)	36.8%	≥ 4 (11), ≤ 1 (227)	4.6%

The *chi-square* test was employed to compare the antimicrobial resistance rate between *intll-positive* and *intll-negative P. mirabilis* isolates. Susceptibility was determined according to the interpretive criteria of the Clinical and Laboratory Standards Institute 2005.

a *p*-value is less than 0.05.

**Table 2:** Resistance phenotype of class 1 integrons and their gene cassettes in76 intl1-positive Proteus mirabilis isolates.

Integrons group (no. of isolates)	Amplicon size of integrons (Kb)	Gene cassette(s)	Resistance phenotype	ERIC type (number)
A single amplicon produced				
1 (1)	3.5	aadB-cat-oxa10-aadA1	KmCAmSmSp	G (1)
2 (1)	3.2	aac(6’)-Ib-aacA7-cmlA	GmCTpSmSp	H (1)
3 (38)	2.1	dfr12-orfF-aadA2	TpSmSp	C (9), D (4), E (2), G(21), H (1), ND(1)
4 (8)	1.8	aadB-aadA2	GmSmSp	B (2), G (4), ND (2)
5 (5)	1.8	dfrA1-aadA1a	TpSmSp	A (1), B (1), G (3)
6 (3)	1.0	aadA1	SmSp	E (1), G (2)
7 (5)	0.9	dfr12	Tp	C (2), D (1), ND(2)
Two or more amplicons produced				
8 (5)	2.1/0.9	dfr12-orfF-aadA2/dfr12	TpSmSp	C (2), D (2), G (1)
9 (3)	2.1/1.8	dfr12-orfF-aadA2/aadB-aadA2	TpSmSp	C (1), F (1), G(1)
10 (2)	2.1/1.8/0.9	dfr12-orfF-aadA2/aadB-aadA2/ dfr12	TpSmSpGm	C (1), G (1)
11 (1)	3.1/2.1/1.8	aadB-cat-aadA1a/dfr12-orfF-aadA2/dfrA1- aadA1a	KmTpSmSp	G (1)
Negative for cassette regions				
12 (4)	0.15	–	–	C (1), D (2), ND(1)

ND, not detected.

Am, ampicillin; C, chloramphenicol; Gm, gentamicin; Km, kenamycin; Tp, trimethoprim; Sm, streptomycin; Sp, spectinomycin.

### 3.2. Prevalence of classes 1 integrons in the urinary isolates

Class 1 integrons were presented in 76 (24.2%) isolates, from 314 urinary isolates of *P. mirabilis* (Table 1). Among 76 isolates, 63 yielded one amplicon, 9 yielded 2 amplicons, and 4 yielded 3 amplicons of different sizes. The amplicon lengths, corresponding to the approximate sizes of the cassette regions, varied from 0.15 to 3.5 kb (Table 2). The presence of class 1 integrons was classified 12 groups by the length and the numbers of amplicons in a single isolate (Table 2). An integron carrying 2.1-kb insert in length was presented in 38 (60.3%) of the 63 isolates yielded one amplicon. This result demonstrated that a widespread distribution of class 1 integrons and their antimicrobial-resistant gene cassettes existed among urinary *P. mirabilis* isolates with ampicillin resistance in the middle Taiwan.

### 3.3. Characteristic of integrons and arrangement of integron gene cassettes

The PCR products of one amplicon were sequenced directly and more than one amplicons were cloned and subjected to DNA sequence and alignment. Primers of p-1 or p-2 and the second or third in cassettes were used mapping the gene cassettes. Most integrons contained 1 or 2 gene cassettes with various configurations and different sizes as indicated in Table 2. We identified 10 different gene cassettes, including 9 cassettes pertaining to antibiotic resistance and 1 cassettes *(orfF)* encoding non-antimicrobial resistance products. The antibiotic resistance genes included those encoding β-lactamase resistance to ampicillin (bla_0XA___10_), dihydrofolate reductase family resistance to trimethoprim *(dfr12),* chloramphenicol resistance genes, *(cmlA, catB),* and aminoglycoside-modifying enzymes family *(aadA1, aadA1a, aadA2, aadB* and *aac(6’)-Ib)* resistance to aminoglycosides. Eight distinct kinds of gene cassette arrays were identified. Of these, the 2.1-kb insert with *dfr12-orfF-aadA2* and 1.8-kb insert with *aadB-aadA2* were found most prevalent. This 2.1-kb insert was also present in isolates yielding two or more amplicons. Sequence analysis of the 150-bp PCR amplicon had not encoded additional antibiotic resistance genes except the basic genetic elements of class 1 integron. The observation that different bacterial species harboured class 1 integrons with *aadA2* and *dfr12* cassettes is a common finding in clinical medicine [22, 23]. Most of the identical drug- resistant gene cassettes in this study were commonly distributed in pathogenic bacteria isolated from human. The cassette array *aadB-cat-oxa10-aadA1* has not been reported previously in *P. mirabilis,* although it is present in *A. baumannii* (accession: DQ288250), *Klebsiella pneumoniae* (accession:HQ880271), *Proteus sp.* IICAZ2 (accession: HQ386837) and *Providencia rettgeri* (accession: KJ488989).

### 3.4. Clonal spread of functional class 1 integron-harbouring *P. mirabilis*

**Fig. 1:**
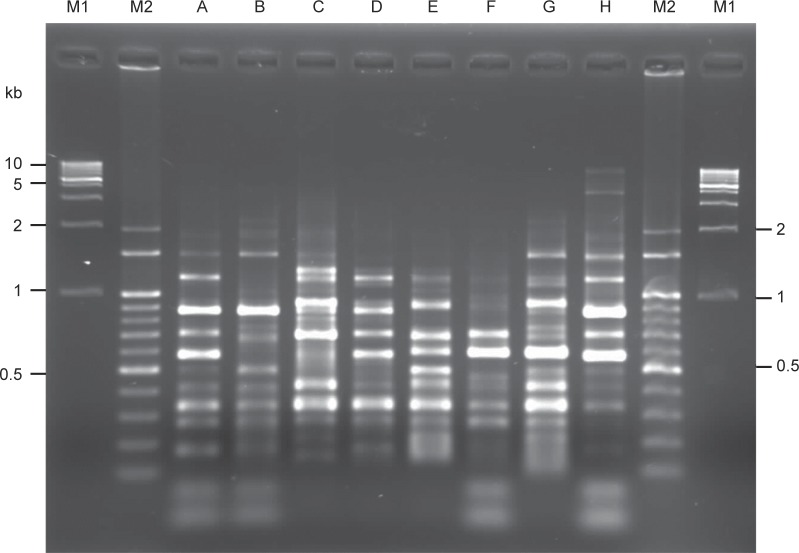
Fingerprinting patterns of eight ERIC types of class 1 integron-positive *P. mirabilis* isolates in this study. Lane M1, 1 Kb molecular size marker. Lane M2, 100 bp molecular size marker. Lanes A-H, fingerprinting patterns of ERIC types A-H, respectively.

ERIC-PCR was used to type the phylogenetic relations of 76 class 1integron-positive *P. mirabilis* isolates. Eight ERIC types were obtained according to the electrophoresis patterns. As shown in fig. 1, products ranging from 200-900 bp were encountered more routinely. From the ERIC-PCR fingerprints results, we found 35 (46.1%, 35/76) integron-positive *P. mirabilis* isolates belonged to ERIC type G. Most of these ERIC type G *P. mirabilis* isolates (71.4%, 25/35) were also positive for class 1 integrons with the *dfr12-orfF-aadA2* gene cassette arrays. This pattern was also reported in urinary *E. coli* isolates both in Taiwan and Korea study [23, 24], and conferring a kind of gene cassettes with stable integration and predominant in multidrug-resistant S. *Choleraesuis* isolates [25]. These results indicated the clonal dissemination of functional class 1 integron harbouring *P. mirabilis* in our hospital.

In conclusion, the class 1 integron-borne gene cassette *dfr12-orfF-aadA2* had been found widely disseminated among *P. mirabilis* isolates from urine samples. The high prevalence of class 1 integrons harboring different arrays of gene cassettes in CTX-M-type ESBLs, including CTX-M-3 and CTX-M-14, within *P. mirabilis* from urine samples which indicates that class 1 integrons were more commonly associated with the *bla*_CTX-M_ gene than non-ESBLs-producing isolates. Furthermore, these functional class 1 integron-harbouring *P. mirabilis* isolates were likely to be the result of clonal spread in our hospital. Additional investigations into class 1 integrons associated ESBL *bla* genes are needed to employ effective means to avoid dissemination of multidrug- resistant bacteria.

## Conflicts of interest statement

The authors declare that there are no conflicts of interest.
